# PReS-FINAL-2005: Prevalence of antinuclear antibodies in schoolchildren across puberty and possible relationship with musculoskeletal pain. A longitudinal study

**DOI:** 10.1186/1546-0096-11-S2-P18

**Published:** 2013-12-05

**Authors:** F Sperotto, S Brachi, M Seguso, F Vittadello, F Zulian

**Affiliations:** 1 Department of Pediatrics, University of Padua, Padua, Italy; 2 Department of Laboratory Medicine, University of Padua, Padua, Italy

## Introduction

Antinuclear antibodies (ANA) are frequently found in children with connective tissue diseases but can be also found in healthy individuals even in absence of autoimmune conditions, with a prevalence ranging from 13.3% (titer ≥1:80) to 5.0% (titer ≥1:160). Puberty is a period of important changing in immune system because of the sexual and adenohypophyseal hormones modulation; in fact many autoimmune and connective tissue diseases have their onset in this period. To date, a few studies have evaluated the role of ANA in healthy subjects but no one has explored their meaning and frequency across the puberty switch.

## Objectives

To evaluate prevalence and persistence of ANA in subjects without any evident autoimmune disease followed for 3 years, and their possible relationship with chronic non-inflammatory musculoskeletal pain (MSP).

## Methods

Each subject underwent a general and rheumatologic examination focusing on presence of chronic non-inflammatory MSP and including the evaluation of the pubertal stage. Chronic MSP was defined as continuous or recurrent pain lasting more than 3 months and heavily interfering with daily activities, according to the International Association for the Study of Pain. Subjects with past of present sign of any neurological, skeletal, metabolic or autoimmune conditions were excluded. Family history for autoimmune diseases in first degree relatives was also investigated. Finally, each subject underwent laboratory tests to determine the presence of ANA, ENA and anti-dsdna, following the international guidelines. Subjects with ANA positivity (titer ≥1:80) and/or MSP have been re-evaluated with the same methods 3 years later.

## Results

261 subjects, aged 8-13 years, entered the study. 32 (12.3%) resulted ANA+, equally distributed as far as gender and pubertal status. None of the ANA+ subjects resulted positive at ENA or anti-dsdna testing. A positive family history for autoimmune conditions was reported in 6.5% of the subjects.

Three years later, in the group of patients followed for MSP (no. 67) ANA-positivity significantly increased from 13.4% to 44.8% (p < 0.001) showing a trend to involve more pre-pubertal subjects than pubertal ones and more females than males, without statistical significance. Particularly, ANA positivity involved more pubertal females than pubertal males (50.0% vs 28.0). In the cohort followed for ANA-positivity (no. 28) 92.9% of subjects confirmed the ANA-positivity 3 years later, showing a significant increase of autoantibodies titer during time (p = 0.002). The prevalence of positive family history did not significantly changed during the study period. None of the ANA+ subjects resulted positive at ENA or anti-dsdna testing. Overall, no significant association between ANA-positivity and MSP was found.

## Conclusion

Prevalence and titer of ANA increase across puberty, especially in females, but have no relationship with MSP. This phenomenon could be explained by the complex hormonal changing of the puberty switch period. Further long-term prospective studies are needed to clarify the potential role of ANA as marker of autoimmune-rheumatic conditions, particularly in this period.

## Disclosure of interest

None declared.

